# Psychiatric autopsy in two cases of assessment testamentary capacity

**DOI:** 10.1192/j.eurpsy.2022.1552

**Published:** 2022-09-01

**Authors:** G. Strete, D. Turliuc, V. Hadareanu, S. Turliuc

**Affiliations:** 1 UMFST TARGU MURES, Psychiatry M4, TARGU MURES, Romania; 2 UMF GRIGORE T POPA, Neurosurgery, IASI, Romania; 3 UMFST TARGU MURES, Forensic Medicine, TARGU MURES, Romania; 4 UMF GRIGORE T POPA IASI, Psychiatry, IASI, Romania

**Keywords:** Testamentary capacity, Mental Capacity, Will contestation, Psychiatric autopsy

## Abstract

**Introduction:**

In Romania, in accordance with current law, “the will is the unilateral, personal and revocable act by which a person named testator disposes, in one of the forms required by law, for the time when he will no longer be alive”. The increasing complexity of modern financial and family structures has led to an increase in testamentary disputes, a fact reflected by the large number of forensic examinations in civil cases.

**Objectives:**

Mental capacity are the majore concerns in the many of issues in elders but the great challenge is its retrospective evaluation, when the patient no longer exists. We focuse on the testator’s mental capacity at the time the will was written.

**Methods:**

We present two cases of will contestation in post mortem, in which the testator’s age was 65, respectively 70 years at the time of executing the will.

**Results:**

In the first case the testator dies one month after he signed the will, the cause of death was cardiorespiratory arrest; cervical neoplasm; In the second case the testator dies two years after he signed the will. In both cases the patients did not have a history of neuropsychiatric disorders in the family doctor’s records. The circumstances of the production of a will, including the mental state of the testators and the true wishes of the testators were reconstructed using a psychiatric autopsy, based on the documents provided.

**Conclusions:**

The complexity and subtlety of the problems reflected in these cases highlights the need to go beyond traditional criteria and assess situation-specific factors.

**Disclosure:**

No significant relationships.

